# Biopesticidal Potential of the *Cannabis sativa* L. Metabolites: A Denoised, Docking-Informed QSAR Model

**DOI:** 10.3390/ijms27188000

**Published:** 2026-09-08

**Authors:** Dinara Karamanova, Nikita Erin, Alexander Bodrov, Varvara Tkachenko, Victor Safronov, Maxim Fedorov

**Affiliations:** 1 A.A. Kharkevich Institute for Information Transmission Problems, Russian Academy of Sciences, Bolshoy Karetny per. 19, Moscow 127051, Russia; nikitaerin20162016@gmail.com (N.E.); bodrovscience@gmail.com (A.B.); varvara.tkachenko.99@gmail.com (V.T.); safronov@phystech.edu (V.S.); 2 AI Institute, ITMO University, 49 Kronverksky Pr., St. Petersburg 197101, Russia; 3 School of Applied Mathematics and Informatics, Moscow Institute of Physics and Technology, 9 Institutskiy per., Dolgoprudny 141701, Russia

**Keywords:** *Cannabis sativa* L., biopesticides, metabolomics, machine learning, molecular docking, artificial intelligence

## Abstract

Plant metabolites are a promising source of new biopesticides, but their chemical diversity exceeds the capacity of experimental screening. *Cannabis sativa* is a particularly attractive crop for discovering such compounds, although its metabolome has not been systematically evaluated for biopesticidal potential. Here, we computationally analyzed 5211 compounds annotated as *C. sativa* metabolites in the Cannabis Compound Database (CCD) using an integrated framework combining graph-based molecular prediction and protein–ligand interaction analysis. Initial prioritization employed a directed message passing neural network (DMPNN) trained on molecular graphs augmented with RDKit descriptors. The DMPNN predictions were integrated with a CatBoost-derived docking-consistency score based on residue-level Vina interaction terms, reducing the false-positive rate by about 60% compared with the structural model alone. Informative ligand-residue interactions were identified using a random matrix theory (RMT) framework. The DMPNN identified 1010 compounds as DMPNN-positive (score ≥0.70), indicating structural characteristics more consistent with the DS2 pesticide reference set than with the DS3 AChE-inactive reference set. Then, these compounds were filtered using annotations from the CCD to retain 44 secondary metabolites. Finally, the 44 compounds were ranked by the final ensemble score. Compared with reference pesticides, *C. sativa* metabolites showed higher predicted median oral LD_50_ values and fewer organ-specific toxicity alerts at the dataset level, although not for all endpoints. Overall, the combined structural and docking-informed workflow identified a small, chemically diverse set of high-ranking *C. sativa* compounds that can now be prioritized for experimental validation.

## 1. Introduction

The intensive use of synthetic pesticides has led to long-term contamination of soils and water bodies, the decline of non-target organisms, and reduced biodiversity. At the same time, pests are rapidly developing resistance to commonly used insecticides, necessitating higher doses and more frequent applications. This creates a vicious cycle: the more pesticides are used, the greater the environmental damage and the faster their effectiveness declines [1].

These challenges are driving a shift toward biopesticides of natural origin. Plant metabolites, unlike their synthetic counterparts, degrade more rapidly, accumulate less in the environment, and exhibit complex modes of action that slow the development of resistance [2].

Industrial hemp (*Cannabis sativa* L.) is of particular interest because its metabolome has been extensively characterized and comprises thousands of structurally diverse specialized metabolites [3]. More than 500 compounds have been experimentally characterized, including cannabinoids, terpenoids, flavonoids, phenolic acids, and lipids [4,5]. Several representatives of these metabolite classes have already demonstrated insecticidal, acaricidal, nematicidal, fungicidal, antibacterial, or repellent activities, highlighting hemp as a promising source of bioactive molecules for crop protection [6,7,8,9,10].

Beyond its chemical diversity, *C. sativa* offers an important practical advantage. Unlike many medicinal or wild plant species, industrial hemp is cultivated on a large scale for fiber, seed, and oil production, generating substantial amounts of leaves, inflorescences, and other non-fibrous biomass that remain underutilized despite being rich in specialized metabolites. Therefore, identifying pesticidal compounds from hemp could simultaneously facilitate the discovery of new biopesticides and promote the valorization of agricultural side streams within a circular bioeconomy.

Despite growing evidence for the biological activity of individual hemp metabolites, previous studies have primarily focused on isolated compounds or crude extracts, leaving the pesticidal potential of the *C. sativa* metabolome as a whole largely unexplored. Systematic experimental evaluation of thousands of metabolites is impractical because of the associated cost and labor. Consequently, computational prioritization combining cheminformatics, molecular docking, and machine learning (ML) provides an efficient strategy for identifying the most promising candidates for subsequent experimental validation. Such an approach is particularly relevant given the increasing demand for environmentally safer crop protection agents and the need to exploit the largely untapped chemical diversity of plant specialized metabolites.

Although individual active compounds are already known, no systematic assessment of the broad chemical space reported for *C. sativa* has yet been carried out to determine how broadly potential biopesticidal candidates are represented within it, which chemical classes contribute most to this space, and how structurally diverse the identified compounds are.

Answering this question by experimental means alone is virtually infeasible. Even with a ready library of metabolites, biological testing of thousands of compounds demands considerable resources and cannot serve as an efficient tool for primary screening. A computational approach is therefore needed: one that first isolates the most promising candidates and then allows experimental efforts to be concentrated on a limited number of compounds.

Modern artificial intelligence (AI) and ML methods provide exactly this capability [11]. Graph neural networks are able to capture complex relationships between molecular structure and biological activity, making them an effective tool for the high-throughput virtual screening of large chemical spaces. The relationship between chemical structure and biological activity is well illustrated by established insecticide classes. The structurally related neonicotinoids imidacloprid and clothianidin both act as modulators of insect nicotinic acetylcholine receptors. Similarly, the pyrethroids permethrin and deltamethrin share voltage-gated sodium channels as their principal molecular target [12,13]. However, structural similarity does not necessarily imply similarity in the mechanism of action. Compounds that are similar in chemical structure may differ substantially in their interactions with target proteins, resulting in a fraction of predictions being inevitably false-positives.

This gives rise to a further methodological problem. When structure-based prediction is used as the sole source of information, it becomes difficult to distinguish genuinely promising compounds from molecules that merely bear a superficial chemical resemblance to known pesticides. Increasing the reliability of computational selection therefore requires an independent source of information that reflects not molecular similarity but the presumed mechanism of interaction.

Therefore, identifying plant-derived molecules with pesticide-reference-like structural characteristics can facilitate candidate prioritization for subsequent experimental validation. In this work, an additional independent line of evidence is obtained by analyzing ligand–protein interactions at the amino acid level. Rather than using molecular docking as a standalone predictor of biological activity, we employ it as a mechanistic validation step to assess whether QSAR-based predictions are supported by plausible protein–ligand interaction patterns. Because the resulting interaction descriptors form a high-dimensional and highly correlated feature space, random matrix theory (RMT)-based filtering is applied prior to model construction to retain robust signals while reducing the influence of noise and redundant features.

Accordingly, the present study provides a systematic assessment of the *C. sativa* compounds represented in the Cannabis Compound Database (CCD) as a potential source of biopesticidal candidates, using a multi-level computational scheme that combines structure-based prediction, mechanistic validation, and toxicological assessment.

## 2. Results

### 2.1. Initial Chemical Space as a Representative Basis for Computational Screening

DS1, representing the CCD-derived *C. sativa* chemical space, was dominated by lipids. In contrast, DS2, comprising registered synthetic and biological pesticides extracted from the BPDB, exhibited a more heterogeneous distribution of chemical superclasses. DS3, comprising AChE-inactive compounds used as the operational reference class for classifier training, displayed a chemical composition distinct from both the CCD-derived *C. sativa* dataset (DS1) and the registered pesticide dataset (DS2). Together, these distributions demonstrate the chemical diversity of the datasets used for model development and virtual screening. Pie charts showing the superclass distributions for each dataset (superclasses accounting for less than 1.3% were grouped as “Others”) are provided in the Supplementary Materials (Figure S1).

**Figure 1:**
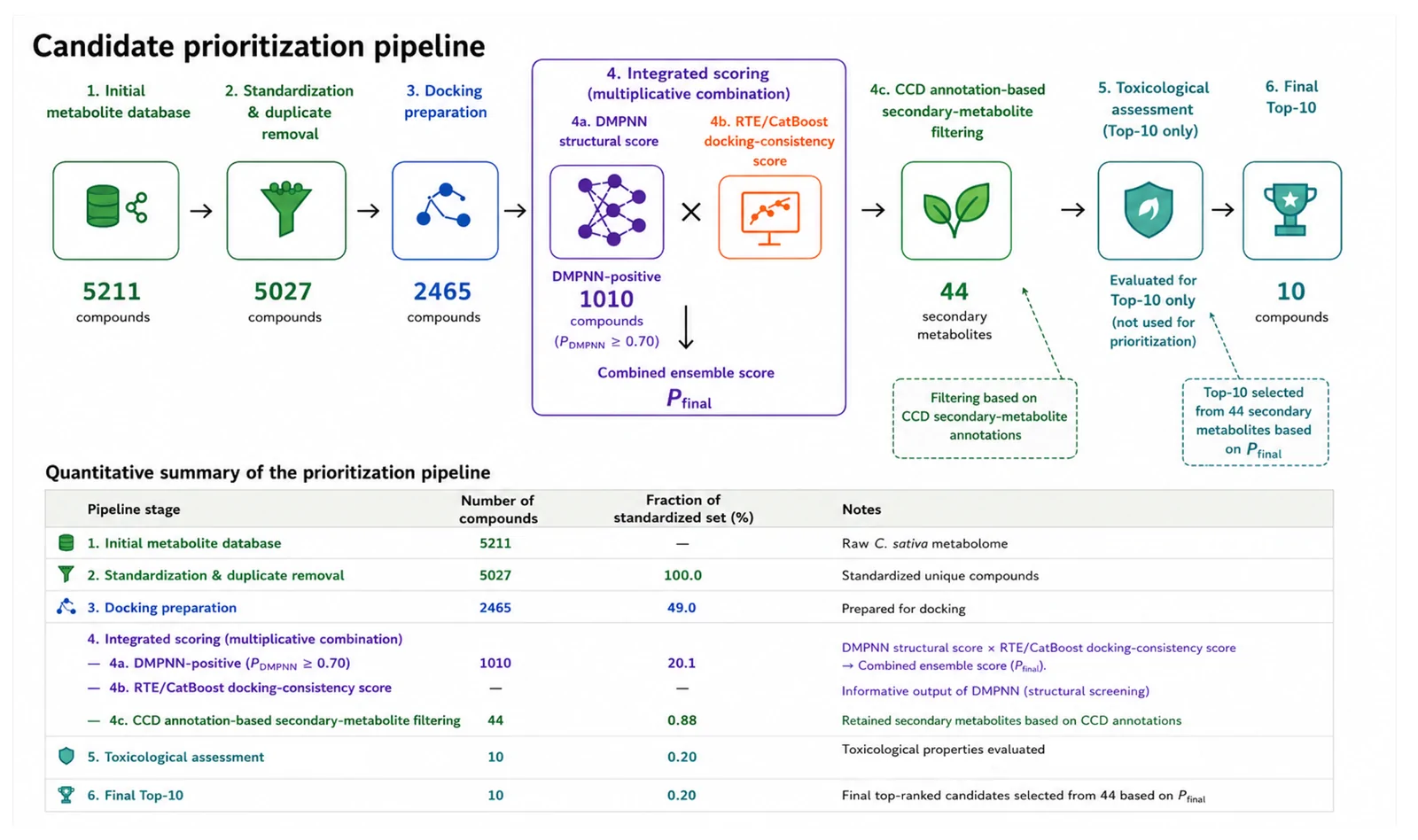
Workflow of the metabolome-scale prioritization of *C. sativa* metabolites. Steps 1–3 describe data curation and docking preparation. In Step 4 the DMPNN structural score and the RTE/CatBoost docking consistency score are combined multiplicatively to obtain the final ensemble score (p); CCD annotation-based filtering retains secondary metabolites. The top-10 are selected from these 44 compounds based on final ensemble score. The steps 5–6 correspond to toxicological assessment and characterization of the final top 10 candidates.

The overall dynamics of candidate space reduction across the successive stages of the computational pipeline are presented in Figure 1. Of the 5211 extracted compounds, 5027 unique structures remained after standardization. The application of the structure-based DMPNN model, the mechanistic Docking-consistency veto, and CCD annotation-based filtering made it possible to reduce the search space to a small number of high-priority candidates for subsequent toxicological assessment and future experimental validation (Figure 1).

### 2.2. Molecular Docking Reveals Target-Specific Differences Between Pesticides and AChE-Inactive Compounds

**Figure 2:**
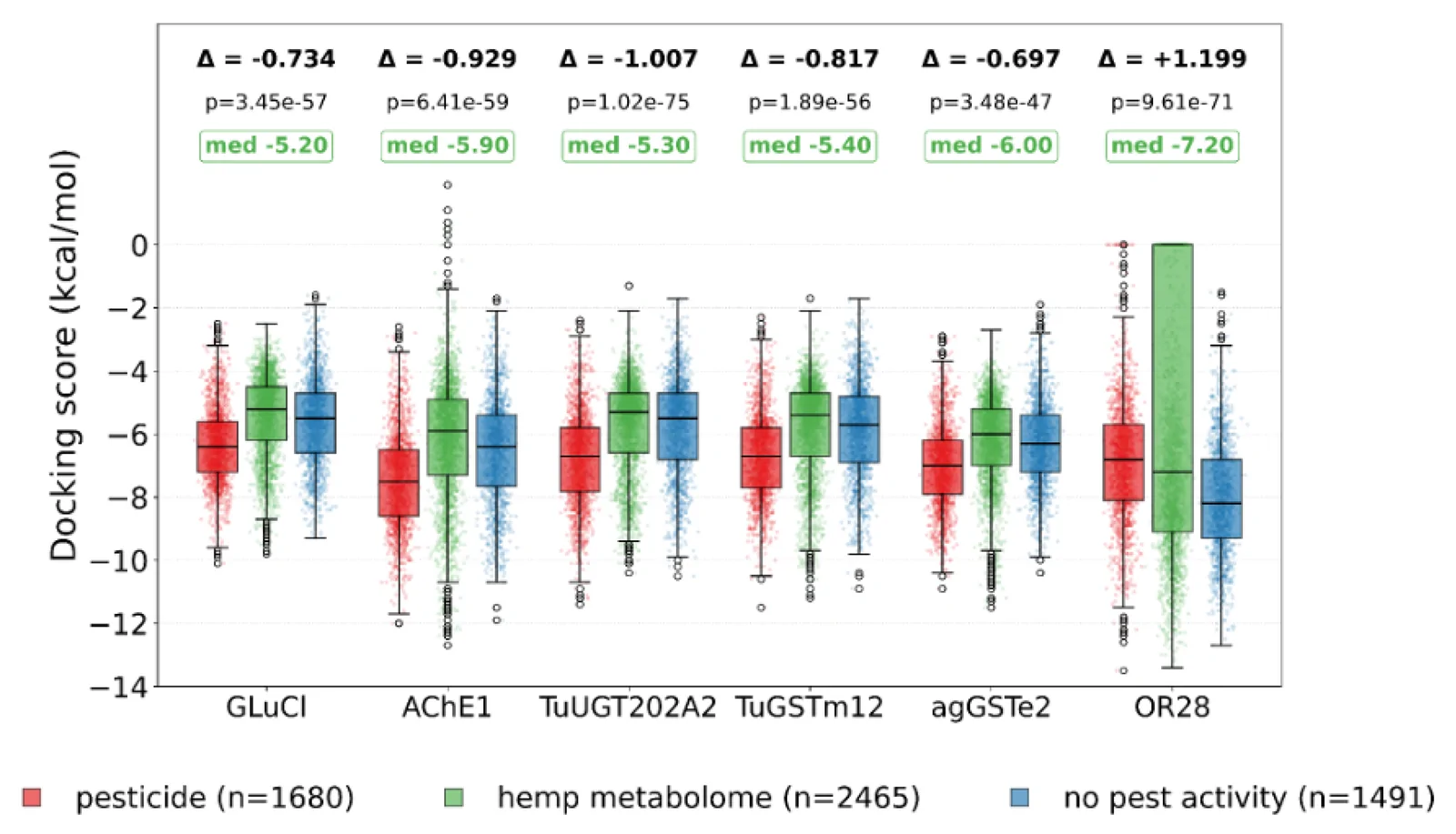
Distribution of docking scores for DS2 pesticides (n=1680), AChE-inactive DS3 compounds (n=1491), and DS1 compounds (n=2465) across six pest-relevant proteins. For five of the six targets, DS2 pesticides showed significantly more favorable docking-derived interaction energies than AChE-inactive DS3 compounds (two-sided Mann–Whitney *U* test (Δ) from −0.70 to −1.01 kcal/mol, Benjamini–Hochberg correction (*p*) ≪0.001), whereas OR28 showed the opposite direction (Δ=+1.20, p≪0.001), illustrating a target-specific contrast within the panel. Median binding energy values of DS1 compounds ranged from −5.2 to −7.2 kcal/mol.

To assess whether the selected protein panel captures interaction patterns associated with the reference pesticide set, molecular docking was performed for ligands successfully obtained from the three datasets (DS1–DS3). Docking was used here to generate residue-level interaction descriptors rather than to estimate absolute binding affinity. The six targets were selected to provide complementary mechanistic coverage, including direct toxicological targets (GluCl and AChE1), proteins involved in xenobiotic metabolism and detoxification (TuUGT202A2, TuGSTm12, and agGSTe2), and the functionally distinct odorant receptor OR28 (Figure 2). Thus, the panel was not intended to represent all possible pesticidal mechanisms, but to provide a diverse target space for evaluating whether candidate metabolites exhibit interaction patterns compatible with the reference pesticide set.

Across five of the six targets, compounds in the DS2 pesticide reference set showed significantly more favorable docking-derived interaction energies than AChE-inactive DS3 compounds, with median energy differences ranging from −0.70 to −1.01 kcal/mol after Benjamini–Hochberg correction (adjusted p<0.001). These target-specific distributions were used as reference profiles for assessing whether candidate metabolites exhibited interaction patterns more consistent with the DS2 pesticide or DS3 operational reference sets.

These results indicate that the selected target panel contains reproducible interaction information that distinguishes the DS2 pesticide set from the DS3 operational reference set. Importantly, this finding does not imply that favorable interaction with all six targets is a universal property of pesticides; rather, the resulting score distributions provide target-specific reference patterns for mechanistic consistency analysis and candidate prioritization.

By contrast, an opposite trend was observed for OR28: AChE-inactive DS3 compounds showed more favorable docking-derived interaction energies than DS2 pesticides (Δ=+1.20 kcal/mol, adjusted p≈0.001). Because OR28 is a chemosensory receptor rather than a canonical toxicological target, this result indicates that favorable interaction with an individual target is not a universal characteristic of pesticide compounds. OR28 therefore serves as a functionally distinct reference within the panel. Its inclusion prevents the mechanistic branch from being interpreted as a simple preference for favorable docking scores across all proteins.

The distribution of DS1 compounds largely overlapped the ranges observed for both reference datasets. Median binding energy values of *C. sativa* metabolites ranged from −5.2 to −7.2 kcal/mol. This overlap indicates that the CCD-derived DS1 chemical space contains compounds spanning interaction profiles observed in both the DS2 pesticide and DS3 operational reference sets. Consequently, docking-derived interaction descriptors are not used as a binary indicator of pesticidal activity. Instead, they provide a target-panel-specific measure of mechanistic consistency. This measure complements the independent structure-based prediction and is used for subsequent candidate prioritization.

### 2.3. RTE Descriptors Provide Complementary Information for Distinguishing the DS2 and DS3 Reference Sets

**Figure 3:**
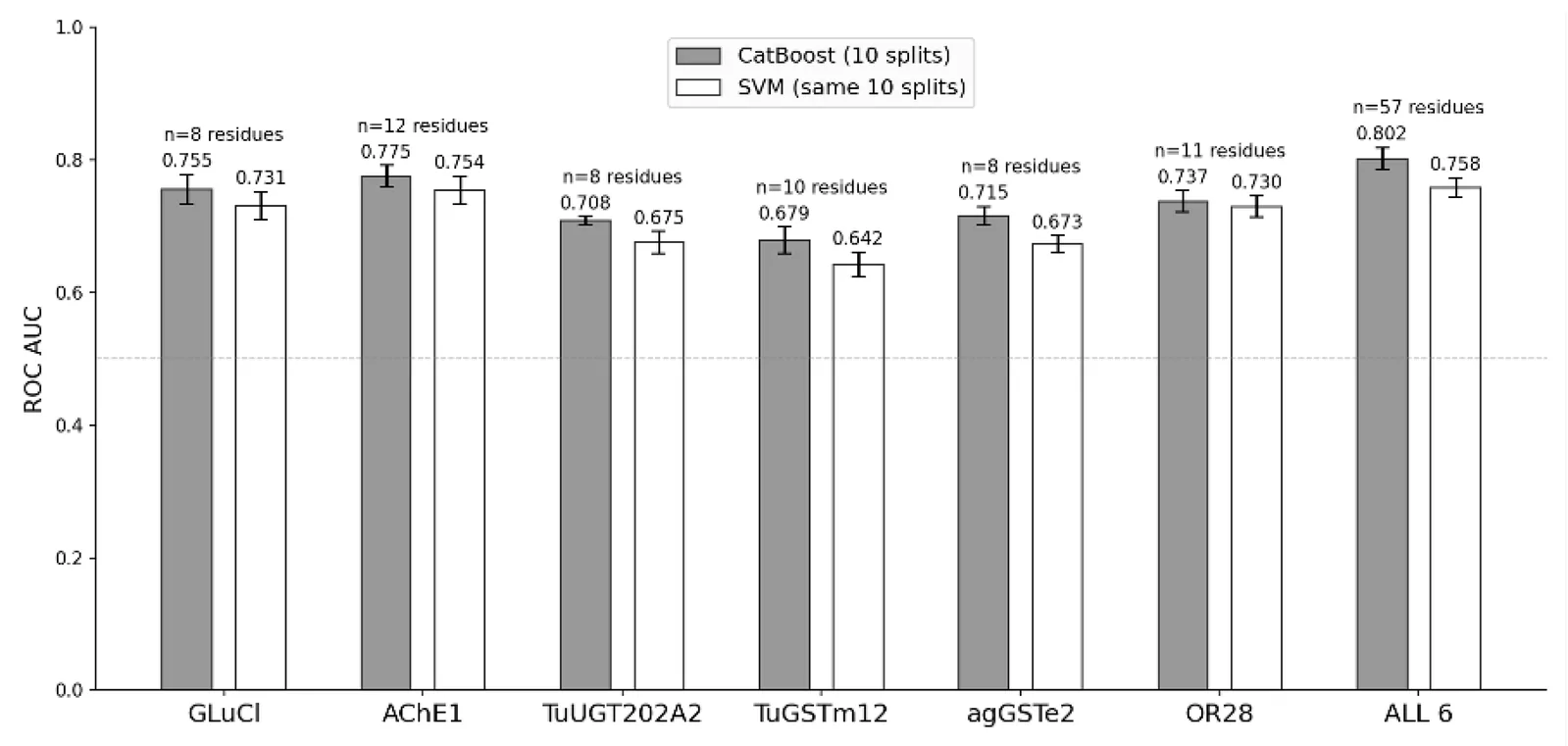
Bar chart of performance metrics for the CatBoost and SVM models across six protein targets and for the combined targets (ALL 6). Error bars represent the standard deviation (SD) based on 10-fold cross-validation. Across all protein groups, both models showed a reproducible discriminative signal separating the DS2 pesticide set from the DS3 operational reference set based on RTE features.

To determine whether residue-level interaction profiles contain predictive information independent of molecular structure, CatBoost and SVM classifiers were trained using RTE-derived interaction descriptors for each of the six target proteins, as well as for their combined feature set (Figure 3). Both algorithms consistently delivered performance exceeding random expectation across all targets, with ROC–AUC values ranging from 0.679 to 0.775 for CatBoost and from 0.642 to 0.754 for SVM. Combining descriptors from all six targets further improved predictive performance, yielding a ROC–AUC of 0.802±0.017.

Although these RTE-descriptor-based models did not reach the accuracy of the structure-based DMPNN classifier (ROC–AUC =0.9283±0.0092, FPR =12.20±1.86%), their performance shows that residue-level interaction signatures contain information that discriminates DS2 pesticides from the DS3 operational reference set. An additional scaffold-based evaluation was performed to assess generalization to structurally different compounds. In the paired comparison, the DMPNN achieved ROC–AUC = 0.9260 and PR–AUC = 0.9393 with the random split, compared with ROC–AUC = 0.9084 and PR–AUC = 0.9297 with the Bemis–Murcko scaffold split (Table S4). Thus, model performance decreased moderately under the more restrictive scaffold split but remained high. Consequently, these descriptors provide an orthogonal data source that complements molecular graph representations.

To exploit the complementary information contained in the residue-level interaction descriptors, CatBoost predictions were incorporated as an asymmetric docking-consistency component. This strategy reduced the FPR to 4.92±1.08% while maintaining a high ROC–AUC (0.9176±0.0093), indicating that the docking-consistency component down-ranks structurally plausible predictions receiving weak support from the selected target-panel interaction profiles.

### 2.4. Ensemble Prioritization Identifies a Chemically Diverse Set of Promising Candidates

After combining the structural score (DMPNN) with the mechanistic information extracted from the RTE descriptors, the AI-assisted framework was used for the final prioritization of *C. sativa* metabolites was performed. Unlike the primary structural screening, the ensemble scheme required consistency between two independent sources of information—structural similarity to known pesticides and correspondence with characteristic interaction profiles with the target proteins. This approach produced a more conservative candidate list, less prone to false-positive predictions.

When interpreting the results, the main ranking metric was the final ensemble score, $p_{\mathrm{final}}$, obtained by multiplying the DMPNN and RTE/CatBoost outputs. For most metabolites, the DMPNN classification score remained below the threshold used for initial structural screening. A total of 1010 metabolites received a DMPNN p≥0.70 and were therefore designated as DMPNN-positive metabolites. This designation refers specifically to the structure-based component of the framework and is distinct from the final ensemble ranking obtained after integration with the RTE-derived docking-consistency score.

Visualization of the chemical space by t-SNE showed that high-scoring metabolites do not form an isolated cluster but are located in regions of partial overlap with the space of known pesticides (Figure S2). At the same time, lipids, sugars, and other primary metabolites are predominantly located outside the regions enriched in high-scoring compounds, whereas monoterpenoids, flavonoids, and alkaloids were more frequently represented among high-scoring regions. These results indicate that the ensemble model assigns high scores to compounds from several chemically distinct secondary-metabolite classes rather than to a single structural class. This observation reflects the distribution of computationally prioritized candidates and should not be interpreted as evidence for the ecological or defensive functions of these metabolite classes in *C. sativa*.

Of the 1010 DMPNN-positive compounds, 44 were retained after CCD annotation-based filtering for secondary metabolites. This filtering step was applied because secondary metabolites are frequently involved in plant chemical defense and include compound classes associated with interactions with herbivores and pathogens [14,15]. These 44 compounds were subsequently ranked by the final ensemble score, $P_{\mathrm{final}}$, and the ten compounds with the highest scores constituted the final candidate set.

**Figure 4:**
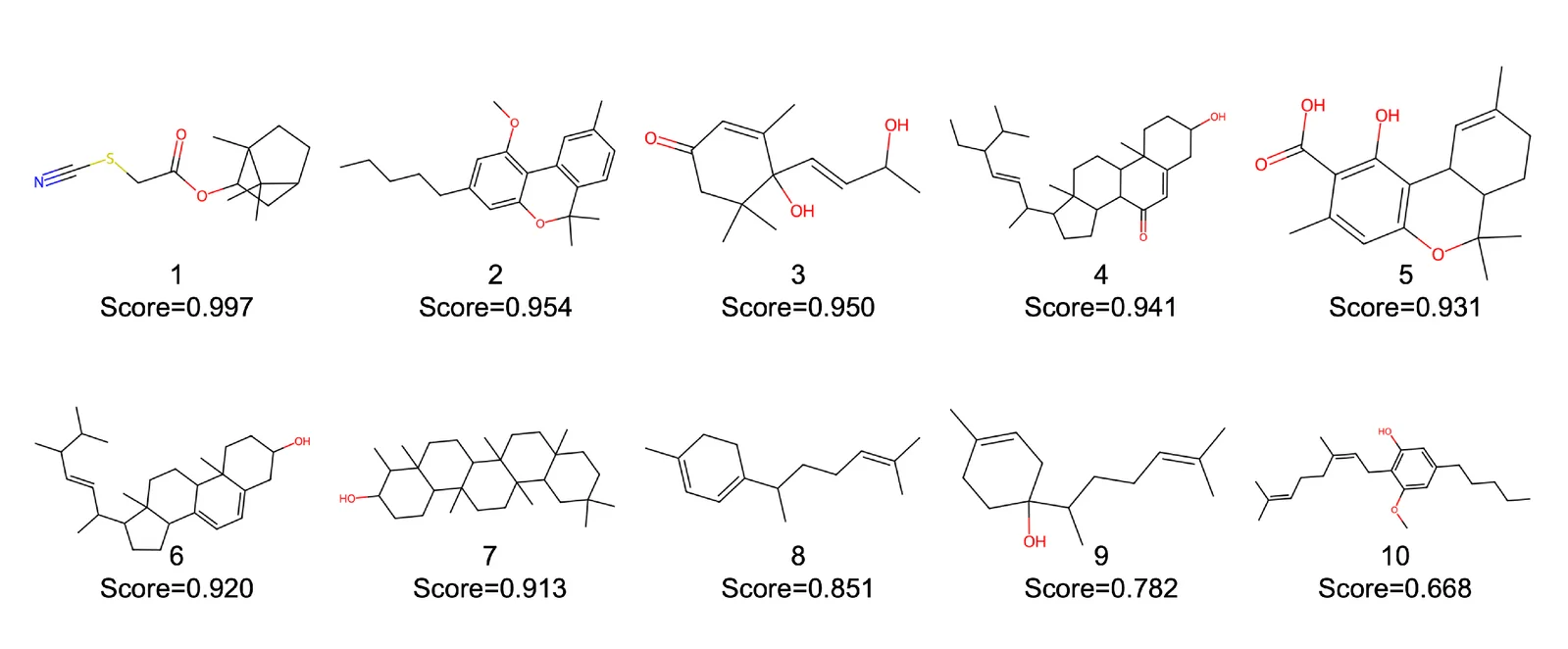
Top 10 *C. sativa* metabolites ranked by the final ensemble score among the 44 secondary metabolites retained after CCD annotation-based filtering of DMPNN-positive compounds: (1) isobornyl thiocyanoacetate; (2) cannabinol methylether; (3) blumenol A; (4) stigmasta-5,22-dien-3β-ol-7-one; (5) Δ-9-tetrahydrocannabiorcolic acid; (6) ergosterol; (7) friedelanol; (8) γ-curcumene; (9) α-bisabolol; (10) cannabigerol monomethylether.

The most promising candidates are shown in Figure 4. Among the top-10 compounds, there are molecules belonging to different chemical classes, including phytocannabinoids, terpenoids, sesquiterpenes, steroids, and triterpenoids. Thus, high-scoring compounds were not restricted to a single chemical class.

The highest-ranked compounds represent several distinct structural solutions associated with pesticide-like chemical space. Isobornyl thiocyanoacetate, the top-ranked candidate, combines a compact bicyclic terpene scaffold with a sulfur- and nitrogen-containing thiocyanate moiety. This distinguishes it from most native hydrocarbon terpenes and may contribute to its close correspondence with structural patterns represented in the pesticide training set. However, the model score alone does not establish which molecular features are responsible for its correspondence with the DS2 pesticide reference class.

Cannabinol methylether and Δ-9-tetrahydrocannabiorcolic acid represent phyto cannabinoid-derived scaffolds characterized by substituted aromatic rings and hydrophobic cyclic or alkyl regions. Blumenol A, γ-curcumene, and α-bisabolol represent oxygenated or hydrocarbon terpenoid structures with substantially lower molecular complexity than the steroid-like candidates. Their occurrence among the highest-ranked compounds indicates that the model did not select a single common scaffold but captured several chemically distinct regions associated with pesticide-like properties.

The steroidal and triterpenoid candidates—stigmasta-5,22-dien-3β-ol-7-one, ergosterol, and friedelanol—share rigid polycyclic frameworks and extended hydrophobic surfaces. Their high ranking may reflect favorable structural and interaction-profile consistency rather than close similarity to conventional small-molecule pesticides. Cannabigerol monomethylether, which received a lower final ensemble score, combines an aromatic cannabinoid core with a flexible prenyl-derived side chain, illustrating how mechanistic filtering can reduce the ranking of structurally plausible compounds when docking-derived support is weaker.

It should be emphasized that the inclusion of a compound in the final list does not imply confirmation of its pesticidal activity. The resulting ranking should be regarded as the outcome of multi-level computational prioritization based on structural and RTE-derived information, followed by toxicological assessment.

### 2.5. Dataset-Wide Toxicological Comparison

Following completion of the computational candidate prioritization and formation of the final top-10 candidate set, we performed a separate toxicological and ecotoxicological assessment. As a first level of this analysis, the complete standardized *C. sativa* metabolite dataset (DS1, n=5027) was compared with the pesticide reference dataset (DS2, n=1709) across 11 predicted toxicological and ecotoxicological endpoints. This dataset-wide comparison was performed to establish the broader toxicological context in which the final candidates could subsequently be interpreted. Toxicological predictions were not used for candidate selection or ranking.

The predicted median acute oral LD_50_ values were higher for *C. sativa* metabolites than for the pesticide reference set. For rats, the median values were 1480 mg/kg for DS1 and 1250 mg/kg for DS2, while the corresponding values for mice were 1290 and 990 mg/kg, respectively. These differences indicate a lower predicted acute oral toxicity of the DS1 chemical space at the dataset level, although the distributions substantially overlapped and individual compounds with unfavorable predictions remained present.

More pronounced differences were observed for selected organ-specific toxicity endpoints. Positive hepatotoxicity and DILI predictions occurred less frequently among *C. sativa* metabolites than among pesticides. In contrast, the proportion of compounds predicted as Ames-positive was higher in DS1, indicating that the DS1 chemical space should not be described as uniformly safer across all endpoints.

The largest difference in aquatic toxicity was observed for the 96-h Fathead minnow endpoint. A low-toxicity prediction was obtained for 86% of *C. sativa* metabolites compared with 29% of pesticides, and the median predicted LC_50_ values were 0.29 and 0.03 mmol/L, respectively. By contrast, the median predicted LC_50_ values for *Daphnia magna* were similar between the datasets (0.03 versus 0.02 mmol/L), suggesting no clear dataset-wide advantage for this endpoint.

The model-applicability estimates provided by Syntelly were examined alongside the endpoint predictions to assess the reliability of the dataset-level comparisons. Model applicability was used as a confidence measure, not as an exclusion criterion. No DS1 or DS2 compounds were removed from the comparisons. For all endpoints except cardiotoxicity, the proportion of predictions outside the corresponding model applicability domain was below 15% in the analyzed datasets (Supplementary Table S5). Cardiotoxicity was a clear exception, with approximately 65% of predictions falling outside the model applicability domain. Given this limited coverage of the relevant chemical space, cardiotoxicity results were retained for completeness but were not used for comparative interpretation. Endpoint-specific applicability statistics for DS1 and DS2 are reported in Supplementary Table S3.

### 2.6. Toxicological Assessment of the Final Candidates

After the final top-10 metabolites had been selected based on the ensemble score and mechanistic consistency, their toxicological profiles were examined individually across the same 11 endpoints used in the dataset-wide analysis (Table 1). For comparative interpretation, the selected metabolites were evaluated against a reference panel of ten widely used synthetic pesticides representing different classes and mechanisms of pesticidal action (Table 2).

This post-prioritization analysis was not used to alter the ranking of candidates following the prioritization process. Instead, it served to assess whether a high ensemble ranking was associated with adverse toxicological characteristics or, conversely, whether the selected candidates exhibited comparatively favorable predicted toxicological profiles based on the modeled endpoints.

For most of the metabolites studied, relatively high predicted acute oral toxicity (LD_50_) values were obtained for both mice and rats, corresponding to the range of moderately or slightly toxic compounds. At the same time, the median LD_50_ values for the final set of metabolites exceeded the corresponding values calculated for the control group of synthetic pesticides, indicating a lower predicted acute toxicity of the prioritized metabolites relative to this reference pesticide panel.

Analysis of individual toxicological endpoints also revealed differences between the two groups of compounds. Among the prioritized metabolites, hepatotoxicity, Ames-test mutagenicity, carcinogenicity, and drug-induced liver injury (DILI) were predicted substantially less frequently than among the selected synthetic pesticides. At the same time, some candidates retained predicted signs of toxicity for certain endpoints, which underscores the need for their subsequent experimental validation. Thus, the toxicological predictions were used to characterize the safety profiles of the already selected candidates and were not used to modify their ranking.

The most pronounced differences between the natural and synthetic compounds were observed when comparing predicted toxicity to aquatic organisms. For some commercial pesticides, extremely low LC_50_ values were obtained, corresponding to a high hazard to aquatic ecosystems, whereas most of the metabolites studied were characterized by higher predicted LC_50_ values and, accordingly, lower acute toxicity to *Daphnia magna* and Fathead minnow. At the same time, some metabolites also exhibited unfavorable predicted aquatic toxicity values; these results were therefore considered when interpreting the safety profiles of the selected candidates, but they did not affect candidate selection or ranking.

Among the compounds studied, cannabigerol monomethylether was characterized by a favorable combination of its ensemble ranking and toxicological profile, showing no signs of mutagenicity, carcinogenicity, hepatotoxicity, or DILI within the prediction models used. However, this assessment reflects exclusively the results of the *in silico* analysis and should be regarded as a basis for subsequent experimental validation rather than as confirmation of the compound’s safety.

To assess whether the toxicological conclusions were dependent on the use of a closed prediction platform, we additionally implemented an open and reproducible version of the published Syntelly modeling workflow as an MCP server. Endpoint-specific model performance was first evaluated using five-fold random cross-validation and subsequently using a more stringent Bemis–Murcko scaffold-disjoint evaluation (Supplementary Table S3). The scaffold-based evaluation generally resulted in lower performance than random cross-validation, as expected when structurally related compounds are prevented from occurring in both training and validation folds, and therefore provides a more conservative estimate of generalization to structurally novel compounds. The open reproduction supported the use of several endpoints for comparative screening, while also identifying endpoints for which predictions require greater caution. In particular, reproductive-toxicity modeling showed limited robustness in the open reproduction and was therefore not used as a major basis for comparative safety conclusions.

### 2.7. Experimental Occurrence Evidence for DMPNN-Positive and Top-Ranked Metabolites

We assessed the level of experimental occurrence evidence among the 1010 DMPNN-positive metabolites, first using the annotation status available in CCD [16]. The CCD distinguishes compounds for which quantitative experimental data are available (“Detected and Quantified”) from those for which such quantitative data are unavailable (“Expected but not quantified”). Among the compounds in the CCD, 151 are classified as “Detected and Quantified”. The 1010 DMPNN-positive metabolites were cross-referenced against these 151 compounds, and 30 metabolites (3.0%) matched entries with the “Detected and Quantified” status. Stereochemical information was not considered during this matching procedure.

The remaining compounds in the CCD are assigned the label “Expected but not quantified,” indicating that quantitative data are not available for them in the database. The absence of quantitative information in the CCD does not by itself demonstrate that a compound is absent from *C. sativa*. Several compounds from the top-10 list illustrate this distinction:

- Δ9-tetrahydrocannabiorcolic acid was reported in a medical cannabis extract as an *m*/*z* 301.15 signal consistent with this compound, although the authors noted that the same signal could also correspond to other constituents [17].
- γ-Curcumene has been identified in the volatile oil of *C. sativa* buds by GC/FID and GC/MS and was also detected in the volatile fraction of commercial hemp cultivars in a later GC-based phytochemical study [18,19].
- Cannabinol methylether was identified in hashish using chromatographic and mass-spectrometric methods, even though reliable tissue-specific quantitative data for this minor cannabinoid are lacking [20].

These examples demonstrate that the absence of a quantitative entry in the CCD does not rule out independent experimental evidence of a compound’s occurrence in the plant.

We therefore treated the “Detected and Quantified” status as an additional layer of experimental support for a compound’s occurrence and abundance in *C. sativa*, rather than as an additional computational screening criterion. Candidates were not excluded from the computational ranking solely because quantitative records were unavailable, since the absence of such data does not establish biological absence. For candidates lacking quantitative or independently reported occurrence data, experimental confirmation of their presence in *C. sativa*, followed by assessment of tissue-specific localization and abundance, should be prioritized before extraction studies and biological testing.

## 3. Discussion

The present study computationally evaluated the CCD-derived *C. sativa* chemical space using a multi-level framework for biopesticide candidate prioritization. These results indicate that the computationally prioritized chemical space spans several secondary-metabolite classes.

A key finding of this study is that combining two independent representations enables more conservative candidate selection. The DMPNN model used molecular graphs and RDKit descriptors to identify structures associated with the pesticide class, whereas the RTE-based model described residue-level ligand–protein interactions obtained from docking. The latter was introduced as an independent check on the structural predictions rather than as an alternative activity predictor. Combining the two models therefore favored compounds supported by both structural and interaction-based information.

The results also indicate that RTE descriptors contain discriminative information complementary to the structure-based model within the DS2/DS3 classification task. Both the linear SVM classifier and the CatBoost ensemble model reliably distinguished DS2 pesticides from the DS3 operational reference set using the same fixed descriptor space (Figure 3), supporting this conclusion. This result supports the use of residue-level interaction profiles as an additional source of information for candidate ranking.

The interpretation of this result depends on how DS3 is defined. DS3 consists of compounds reported as inactive against AChE, but AChE inactivity does not exclude pesticidal activity through other molecular targets. The RTE classifier therefore distinguishes DS2 pesticides from this operational reference set; it is not a general pesticide/non-pesticide classifier. A more rigorous test would require a sufficiently large, experimentally validated, and chemically matched negative dataset covering different pesticidal mechanisms and biological endpoints.

The six-protein target panel imposes a second constraint. These proteins represent complementary biological processes but do not cover the full range of known pesticidal mechanisms. A compound acting primarily through a target absent from the panel may receive weak RTE support despite having pesticidal activity. Extending the panel to additional target classes would increase the mechanistic coverage of the approach.

Other limitations arise from ligand preparation and docking. A substantial fraction of DS1 could not be prepared for docking. Therefore, conclusions based on docking and RTE descriptors apply only to the successfully prepared subset and should not be extrapolated to the complete standardized DS1 dataset. The removal of stereochemical labels is an additional limitation, particularly for natural products with multiple stereogenic centers, because stereochemistry-dependent protein–ligand interactions may not be captured in the docking/RTE analysis. In addition, rigid-receptor docking does not account for receptor flexibility, induced fit, or solvent-mediated effects. The residue-term energies used in this study are therefore numerical descriptors of ligand–protein complementarity rather than physically accurate interaction energies. Molecular dynamics simulations of the prioritized complexes could provide a more detailed assessment at the next stage, when the number of candidates is sufficiently small for per-complex analysis.

The toxicological models give predictions for specific endpoints. Model-applicability estimates were used to judge how far each prediction can be trusted, but were not used to filter compounds. Coverage is uneven across endpoints, and cardiotoxicity in particular is poorly covered. The different components of the framework provide distinct levels of evidence. DMPNN and the ensemble provide computational classification and ranking, whereas RTE descriptors characterize predicted interaction profiles for the selected target panel. None of these outputs constitutes experimental evidence of pesticidal efficacy or safety. The analysis also does not establish metabolite abundance, a defensive role in the plant, ecological function, or synergistic interactions.

Occurrence data provide a separate source of experimental information. Experimental support for occurrence varies across the computationally selected compounds, ranging from quantitative CCD records for a subset of DMPNN-positive metabolites to qualitative analytical reports for several top-ranked candidates. The present study did not determine tissue-specific abundance, extraction yield, seasonal variation, or the economic feasibility of isolation. These factors are important for experimental follow-up but were not used as computational screening criteria.

The chemical diversity of the highest-ranked compounds is informative with respect to the behavior of the ensemble model. The candidate set includes compact terpenoid derivatives, phytocannabinoid-related structures, flexible sesquiterpenes, and rigid steroidal or triterpenoid frameworks. Candidate selection was therefore not dominated by a single scaffold. Different structures may satisfy the structural and RTE-based criteria through different combinations of hydrophobicity, heteroatom distribution, and residue-level interaction patterns. These patterns provide a chemical interpretation of the ranking but should not be taken as experimentally validated pharmacophores or mechanisms of action.

The dataset-wide toxicological comparison provides an additional context for candidate prioritization. At the dataset level, DS1 compounds showed higher predicted median oral LD_50_ values and a lower frequency of several organ-specific toxicity alerts than the pesticide reference set. A particularly marked difference was observed for the Fathead minnow endpoint. However, this trend was not uniform across all endpoints: predicted *Daphnia magna* toxicity was broadly comparable between the datasets, and Ames-positive predictions were more frequent among the metabolites. Therefore, the results do not support a general claim that natural origin implies safety. Rather, within the endpoints and applicability domains of the models used here, the DS1 chemical space showed a heterogeneous set of predicted toxicological profiles, with comparatively favorable predictions for some endpoints but not others.

The origin of the candidates from an established agricultural crop may also be relevant to their practical development. Industrial hemp production generates non-fibrous biomass, including leaves, floral residues, and other processing side streams containing specialized metabolites. Candidate prioritization could therefore help identify compounds in these fractions that warrant experimental investigation. Whether such compounds can be recovered at useful yields and acceptable cost remains to be determined.

For candidates lacking prior experimental evidence of occurrence, validation should first confirm their presence in relevant *C. sativa* tissues. For candidates with reported occurrence, tissue-specific abundance should then be quantified. Subsequent studies should determine extraction yields and assess the feasibility of compound isolation. Candidates available in sufficient quantities can then be tested in target-specific biochemical assays and dose–response pesticidal bioassays. Compounds showing activity should subsequently be evaluated for toxicity to relevant non-target organisms and investigated for their mechanisms of action. Tissue-specific abundance, seasonal variability, extraction yield, and economic feasibility were not evaluated in the present study and will require separate experimental assessment.

## 4. Materials and Methods

### 4.1. Study Design

The workflow integrated two complementary sources of information: (i) a structure-based DMPNN [21] model using molecular graphs and RDKit [22] descriptors and (ii) a docking-derived representation based on residue-term interaction energies. RMT was applied to reduce noise in the interaction feature space before CatBoost [23] classification. Final candidate prioritization combined predictions from both models.

### 4.2. Data Sources

Three datasets were used to build the computational model:

1. DS1 (n=5211) comprised compounds annotated as *C. sativa* metabolites in the Cannabis Compound Database (CCD) [16] and was used exclusively as the discovery set for candidate prioritization. Database inclusion was not considered equivalent to independent experimental confirmation of occurrence, tissue-specific abundance, isolation feasibility, or laboratory availability.
2. DS2 (n=1709)—registered synthetic and biological pesticides extracted from the BPDB and PPDB [24]—served as the positive class for model training.
3. DS3 (n=1698)—compounds from PubChem [25] classified as inactive against acetylcholinesterase (AChE) (BioAssay AID 1347395)—served as the negative class for model training. DS3 was used as an operational negative class rather than as a dataset of compounds demonstrated to lack pesticidal activity. This choice reflects the lack of a sufficiently large and experimentally validated dataset of non-pesticidal compounds suitable for comparison with the DS2 dataset. AChE inactivity nevertheless provides a biologically relevant contrast for a substantial fraction of DS2 pesticides associated with AChE-mediated mechanisms.

All datasets were standardized, including the removal of duplicates, salts, and stereochemical labels, charge neutralization, and SMILES canonicalization. Stereochemical labels were removed because stereochemical information was not consistently available across the source datasets and the primary DMPNN screening was based on a uniform 2D molecular representation. Following standardization, exact structural overlap among DS1, DS2, and DS3 was assessed using canonical SMILES and independently verified using InChI identifiers. No identical structures were detected between DS1 and DS2, DS1 and DS3, or DS2 and DS3; therefore, no additional cross-dataset exclusions were required before model training. The number of compounds in the datasets decreased to 5027 in DS1, 1709 in DS2, and 1694 in DS3. Chemical taxonomic annotation of the compounds was obtained using the NPClassifier tool (v1.6) [26].

### 4.3. DMPNN Classification of DS2 and DS3 Reference Compounds

Primary structure-based prioritization was performed using DMPNN classifier trained to distinguish DS2 pesticides from the DS3 AChE-inactive operational reference set. DMPNN is a directed graph neural network with edge-based message passing (Chemprop [21]), trained on a molecular graph enriched with RDKit descriptors. The model was trained using the optimized hyperparameters summarized in Supplementary Methods Table S1. The training set was randomly split into training, validation, and test subsets. To assess model generalization to structurally different compounds, an additional validation was performed using a Bemis–Murcko scaffold split, in which compounds sharing the same scaffold were kept within the same data partition. The DMPNN was evaluated under both random and scaffold-based splitting using the same model configuration. The results of this additional comparison are reported in Supplementary Materials (Table S4) For each metabolite, the model produced a classification score reflecting its correspondence with the DS2 pesticide reference class relative to the DS3 operational reference class. Compounds with a score of p≥0.70 were designated as DMPNN-positive and retained for subsequent prioritization.

### 4.4. Molecular Docking

Molecular docking was performed using Vina-GPU 2.0 [27] with logging of ligand binding affinity estimates for each amino acid residue of the active site (Residue-Term Energy). Docking was performed solely to generate residue-level interaction descriptors for the mechanistic branch of the workflow described above. Accordingly, docking scores and poses are not interpreted here as estimates of binding affinity or as descriptions of binding modes; they are used only as a uniform numerical featurization of protein–ligand complexes.

For each of the six targets, the distributions of compound binding energies were compared using the two-sided Mann–Whitney *U* test. Correction for multiple comparisons was applied using the Benjamini–Hochberg method (FDR control). Effect size was estimated as the median difference Δ=med(DS2)−med(DS3); a value of Δ<0 corresponds to more favorable docking-derived interaction energies for DS2 than for DS3. The difference was considered significant at adjusted p<0.001.

#### 4.4.1. Ligand Preparation

Due to difficulties in generating correct three-dimensional conformations and in parameterization, 2704 ligands were excluded from further consideration. This predominantly concerned molecules with high conformational flexibility and a large number of rotatable bonds (glycerolipids, glycerophospholipids, and fatty acid esters). Only 494 of 2084 glycerophospholipids (23.7%) and 104 of 1104 glycerolipids (9.4%) were retained. Together, these two lipid classes decreased from approximately 63.4% of the standardized DS1 to 24.3% of the successfully prepared subset (Figure S1). Consequently, the docking- and RTE-based analyses apply only to the successfully prepared DS1 subset (n=2465) and should not be extrapolated to the complete metabolite dataset.

#### 4.4.2. Selection of Target Proteins

Ultimately, 5636 ligands were prepared (2465 for DS1, 1680 for DS2, and 1491 for DS3). Six target proteins (Table 3) were used as objects for molecular docking. The panel was not intended to represent the full mechanistic diversity of pesticides, but to provide a functionally diverse reference space for evaluating residue-level interaction patterns.

Five target structures—GluCl, TuUGT202A2, TuGSTm12, agGSTe2, and OR28—were obtained from the PDB database; the acetylcholinesterase structure AChE1 was taken from the AlphaFold database. Protein structures were prepared using standard PyMOL v3.1.3 [28] and Meeko [29] workflows including solvent removal, protonation, charge assignment, and conversion to PDBQT.

### 4.5. RTE Descriptors

For each protein–ligand complex, a Residue-Term Energy (RTE) feature profile was generated. This profile describes ligand interactions with individual amino acid residues in greater detail than conventional integral binding-energy estimates.

Residue-resolved descriptions of protein–ligand complexes are conceptually related to protein–ligand interaction fingerprints, which encode contacts between ligands and binding-site residues and have been used for docking post-processing and virtual screening [30]. Similar fingerprint-based interaction analyses have also been applied to plant-derived natural products, including essential-oil compounds from sunflower receptacles [31]. In the study, we use the same general principle of residue-resolved interaction representation, but replace binary contact fingerprints with quantitative residue-term energy descriptors and apply RMT filtering to select a compact, noise-reduced amino-acid set.

RTE descriptors were arranged as separate residue-energy matrices for each protein and filtered using Random Matrix Theory (RMT) before model construction. RMT was applied as a training-set-fitted procedure for denoising and residue selection based on the Marchenko–Pastur separation of random and structured correlation components [32,33]. Its application to protein–ligand data follows the framework of Lee, Brenner, and Colwell [34]. For each outer train/test split and each protein, residues were ranked on the training set by their RMT score $s_{i}$. The optimal number of ranked residues, $m^{*}$, was selected by inner cross-validation using the cumulative energy of the top-*m* residues. Within the resulting top-$m^{*}$ set, the final amino-acid residues were defined as the smallest train-only subset accounting for 98% of the energetic contribution. The selected original residue-energy columns, rather than reconstructed RMT features, were then used as the mechanistic RTE descriptor space for CatBoost classification. The full residue-selection protocol is described in Appendix A.

### 4.6. Analysis of Mechanistic Information

To assess the informativeness of the RTE descriptors, a CatBoost model was employed. The task consisted of testing whether the RTE profiles contained sufficient information to distinguish DS2 pesticides from the DS3 operational reference set. To verify the robustness of the detected signal, a linear Support Vector Machine (SVM) classifier was additionally used. Both architectures are robust to class imbalance [35]. Agreement between the results obtained by different classifiers was considered evidence that the discriminative information resides within the RTE descriptors themselves, rather than being an artifact of the specific ML algorithm.

### 4.7. Ensemble Prioritization of Candidates

The final ensemble score was calculated as the product of the DMPNN classification score and the CatBoost score obtained from the RTE descriptors:

$$p_{\mathrm{final}}=p\left(\mathrm{DMPNN}\right)\times p\left(\mathrm{RTE}\right)$$

This multiplicative combination was motivated by the product rule used in classifier fusion [36]. In the present workflow, the product was used as a conservative docking-consistency rule rather than as a calibrated joint posterior probability. A high final score was therefore obtained only when both the structure-based and RTE-based models supported assignment to the pesticide class.

Performance was assessed using ROC–AUC, PR–AUC, and the false positive rate (FPR). For calculation of the FPR, a classification threshold of 0.5 was used. ROC–AUC and FPR were summarized across cross-validation folds by their mean and standard deviation. The 0.5 threshold was used only for performance evaluation and was not applied as a hard cutoff during metabolite ranking.

### 4.8. In Silico Assessment of Acute, Organ-Specific Toxicity and Ecotoxicity Profiles of Metabolites and Comparison of Toxicological Profiles

Toxicological assessment was performed after completion of the computational candidate prioritization and formation of the final top-10 candidate set. Toxicity predictions therefore were used as a subsequent safety-oriented assessment of the prioritized compounds.

Eleven toxicological and ecotoxicological endpoints were evaluated using the Syntelly platform [37]: acute oral LD_50_ (rats, mice); organ-specific toxicity—Ames test, carcinogenicity, hepatotoxicity, DILI, cardiotoxicity, reproductive toxicity; and ecotoxicity—*Daphnia magna* LC_50_ (48 h), 96-h Fathead minnow LC_50_, and acute aquatic toxicity classes derived from predicted aqueous LC_50_ values.

Table S2 reports the cross-validated performance published for these models. RMSE was used for regression endpoints and ROC-AUC for classification endpoints. At the metabolome-wide level, the predicted toxicological profiles of all 5027 *C. sativa* metabolites (DS1) were compared with those of the complete pesticide reference set (DS2, n = 1709). For regression endpoints, the distributions were summarized using medians; for classification endpoints, the proportions of compounds predicted as positive were calculated.

For each compound–endpoint prediction, the model-applicability estimate provided by Syntelly was recorded and used to assess the reliability of endpoint-level interpretation. Model applicability was not used as an exclusion criterion, and no compounds were removed from the dataset-level comparisons on the basis of an applicability cutoff. Instead, the proportion of compounds outside the applicability domain was calculated separately for each endpoint and dataset and is reported in Supplementary Table S5. For most endpoints, fewer than 15% of compounds were outside the corresponding model applicability domain. Cardiotoxicity represented a notable exception, with approximately 65% of compounds falling outside the model applicability domain; consequently, cardiotoxicity predictions are reported for completeness but were not used for comparative toxicological interpretation. Predicted LD50 and LC50 values were treated as approximate model-based estimates suitable for comparative screening rather than as experimentally determined toxicity values.

To check the reproducibility of this workflow, we also implemented an open version of the published modelling strategy [38] on publicly available toxicological data [39]. Model performance was evaluated using five-fold random cross-validation, with ROC–AUC for classification endpoints and RMSE for regression endpoints. To assess generalization to structurally distinct compounds, the open models were additionally evaluated using a Bemis–Murcko scaffold-disjoint split. Random and scaffold-based validation results are reported separately in Supplementary Table S3. The open MCP implementation was used as a reproducibility and robustness analysis and did not replace or redefine the model-applicability estimates provided by Syntelly.

In addition, the toxicological profiles of the final top-ranked metabolites were compared with a reference panel of ten widely used synthetic pesticides (Table 4) acting on octopamine, ryanodine and GABA receptors, acetylcholinesterase, and C14-demethylase. This second analysis was used for candidate-level interpretation rather than for characterization of the entire chemical space.

## 5. Conclusions

In this study, we carried out a systematic AI-assisted computational assessment of the *C. sativa* compounds represented in the Cannabis Compound Database in order to identify compounds showing structural and interaction-profile characteristics similar to the DS2 pesticide reference set. The proposed ensemble computational pipeline combined structural information with a mechanistic representation of the protein–ligand interactions. Integrating these two independent sources of evidence enabled a more conservative and better-substantiated approach to candidate prioritization. The ML-based computational ranking identified a chemically diverse set of CCD-derived *C. sativa* compounds with high ensemble scores across several structural classes. We propose these compounds as candidates for experimental testing rather than as compounds with demonstrated pesticidal activity. Because these candidates originate from an established industrial crop, their further investigation may support the higher-value use of underutilized hemp biomass. However, the feasibility of such applications remains to be established. Key factors include metabolite abundance, extraction yield, isolation costs, and scalability. The dataset-wide comparison indicated comparatively favorable ML-predictions for several acute and organ-specific toxicity endpoints relative to the pesticide reference set. However, these differences were endpoint-specific and do not imply that natural origin is a proxy for safety. Future studies should therefore focus on experimental validation of the prioritized compounds and assessment of their feasibility for practical biopesticide development.

## Figures and Tables

**Table 1:** Data on acute toxicity for rats and mice (LD_50_, mg/kg), mutagenicity (Ames test), carcinogenicity, hepatotoxicity, DILI, cardiotoxicity, reproductive toxicity, and toxicity to aquatic organisms (*Daphnia magna* and Fathead minnow, LC_50_, mmol/L) for the top 10 computationally prioritized *C. sativa* metabolites.

No.	Mouse Oral LD_50_ mg/kg	Rat Oral LD_50_ mg/kg	Ames Test	Carcinog.	Hepatotox.	DILI	Cardiotox.	Reprod. Tox.	*Daphnia Magna* LC_50_ mmol/L	96 h Fathead Minnow LC_50_ mmol/L	Acute Aquatic Toxicity 57455-2017
1	313	1000 *	Nontoxic	Nontoxic	Toxic	Toxic	Nontoxic	Toxic	<0.001 *	0.047	Hazard class 3
2	2826	3321	Nontoxic	Nontoxic	Toxic	Toxic	Nontoxic	Toxic	0.004	0.007	Hazard class 2
3	355	1866	Toxic	Nontoxic	Nontoxic	Nontoxic	Nontoxic	Toxic	0.009	0.17	Hazard class 3
4	1764	1391	Nontoxic	Nontoxic	Nontoxic	Nontoxic	Nontoxic	Toxic	0.002	0.035	LC50 > 100 mg/L
5	1388	1467	Nontoxic	Nontoxic	Nontoxic	Nontoxic	Nontoxic	Toxic	0.007	0.006	Hazard class 2
6	908	139	Nontoxic	Nontoxic	Nontoxic *	Nontoxic	Nontoxic	Toxic	0.003	0.041	LC50 > 100 mg/L
7	1502	4636	Nontoxic	Nontoxic	Nontoxic	Nontoxic	Nontoxic	Toxic	0.019	0.025	Hazard class 2
8	2053	3719	Nontoxic	Nontoxic	Nontoxic	Nontoxic	Nontoxic	Toxic	0.002	0.035	LC50 > 100 mg/L
9	1376	3821	Nontoxic	Nontoxic	Nontoxic	Nontoxic	Nontoxic	Toxic	0.004	0.013	Hazard class 2
10	2742	3309	Nontoxic	Nontoxic	Nontoxic	Nontoxic	Nontoxic	Toxic	0.004	0.012	Hazard class 2

* The data are taken from the TOXRIC database.

**Table 2:** Data on acute toxicity for rats and mice (LD_50_, mg/kg), mutagenicity (Ames test), carcinogenicity, hepatotoxicity, DILI, cardiotoxicity, reproductive toxicity, and toxicity to aquatic organisms (*Daphnia magna* and Fathead minnow, LC_50_, mmol/L) for 10 widely used synthetic pesticides: (1) amitraz, (2) tetraniliprole, (3) chlorantraniliprole, (4) flubendiamide, (5) endosulfan, (6) aldicarb, (7) carbosulfan, (8) malathion, (9) parathion, (10) propiconazole.

No.	Mouse Oral LD_50_ mg/kg	Rat Oral LD_50_ mg/kg	Ames Test	Carcinog.	Hepatotox.	DILI	Cardiotox.	Reprod. Tox.	*Daphnia magna* LC_50_ mmol/L	96 h Fathead Minnow LC_50_ mmol/L	Acute Aquatic Toxicity 57455-2017
1	1085 **	800 **	Toxic	Toxic	Toxic	Toxic	Nontoxic	Toxic	0.002	0.012	Hazard class 1
2	1224	1292	Nontoxic	Nontoxic	Toxic	Toxic	Nontoxic	Toxic	0.078	0.097	Hazard class 2
3	1460	1518 **	Nontoxic	Nontoxic	Toxic	Toxic	Nontoxic	Toxic	0.049	0.041	LC50 > 100 mg/L
4	2743	1865 **	Nontoxic	Nontoxic	Toxic	Toxic	Nontoxic	Toxic	0.025	0.065	Hazard class 3
5	7.4 **	18 **	Nontoxic	Nontoxic *	Toxic	Nontoxic	Toxic	Toxic	0.001 *	<0.001 *	Hazard class 1
6	60	31	Toxic	Nontoxic	Toxic	Toxic	Nontoxic	Toxic	0.004	0.008	Hazard class 2
7	74 **	51 **	Nontoxic	Nontoxic	Nontoxic	Nontoxic	Nontoxic	Toxic	0.006	0.006	Hazard class 2
8	412 **	592^**^	Nontoxic	Nontoxic *	Nontoxic	Toxic	Nontoxic	Toxic	0.074 *	0.042 *	Hazard class 3
9	5 **	2 **	Nontoxic	Nontoxic *	Toxic	Toxic	Nontoxic	Toxic	<0.001 *	0.006 *	Hazard class 2
10	1490 **	550 **	Nontoxic	Toxic	Toxic	Toxic	Nontoxic	Toxic	0.019 *	0.012	Hazard class 2

* The data are taken from the TOXRIC database. ** The data are taken from the CompTox database.

**Table 3:** Selected target proteins for molecular docking.

Protein Name	Designation	PDB ID	Organism	Ligand	Cofactor
Glutamate-gated chloride channel (GluCl)	GluCl	3RIF	*Caenorhabditis elegans* (+ Fab *Mus musculus*)	IVM, GLU, CL, NAG, LMT, OCT, UND	—
Carboxylic ester hydrolase/Acetylcholinesterase	AChE1	AF-D8V7J0 (*AlphaFold*) (25)	*Tetranychus urticae*	—	No
UDP-glycosyltransferase 202A2	TuUGT202A2	8SFY	*Tetranychus urticae*	UDP-glucose (UPG)	No
Glutathione S-transferase mu-class	TuGSTm12	8UDB	*Tetranychus urticae*	—	Glutathione (GSH)
Glutathione S-transferase epsilon-class	agGSTe2	2IMI	*Anopheles gambiae*	—	Glutathione (GSH)
Odorant receptor	OR28	8V3D	OR28: *Anopheles gambiae*; Orco: *Apocrypta bakeri*	2,4,5-trimethylthiazole (A1AFC)	No

**Table 4:** List of 10 synthetic pesticides acting on octopamine, ryanodine, and GABA receptors, acetylcholinesterase, and C14-demethylase.

No.	Pesticide	SMILES	Class	Target
1	Amitraz	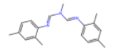	Triazopentadiene	Octopamine receptor modulator
2	Tetraniliprole	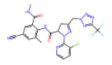	Secondary carboxamide	Ryanodine receptor modulator
3	Chlorantraniliprole	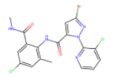	Anthranilamide	Ryanodine receptor modulator
4	Flubendiamide	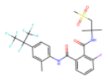	Highly substituted phthalic acid diamide	Ryanodine receptor modulator
5	Endosulfan	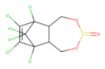	Organochlorine	GABA receptors
6	Aldicarb	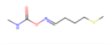	Carbamate	Acetylcholinesterase
7	Carbosulfan	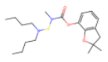	Carbamate	Acetylcholinesterase
8	Malathion	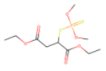	Organophosphate pesticides	Acetylcholinesterase
9	Thiophos (parathion)	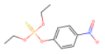	Organophosphate pesticides	Acetylcholinesterase
10	Propiconazole	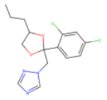	Triazole	C14-demethylase

**Figure A1:**
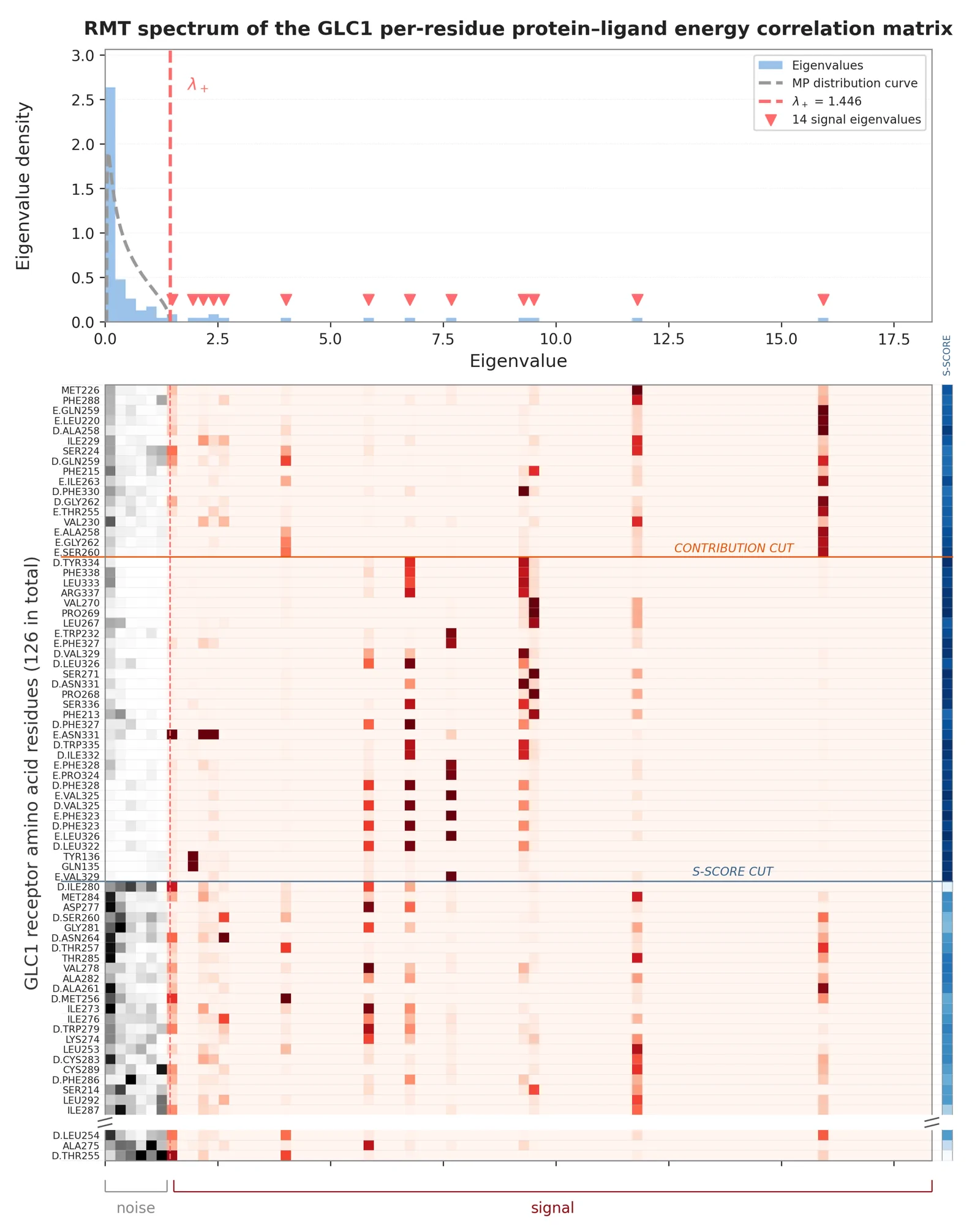
Representative RMT residue-selection diagnostic for GLC1. The (**upper panel**) shows the eigenvalue spectrum of the residue-energy correlation matrix together with the Marchenko–Pastur bulk and the upper threshold $\lambda_{+}$; eigenvalues above this threshold are treated as signal components. The (**lower panel**) shows residue-wise mean squared eigenvector loadings across eigenvalue bins. Gray shades indicate the noise-dominated part of the eigenvalue spectrum, whereas amber-to-red colors indicate eigenvalues associated with signal components. The S-score column summarizes the RMT score $s_{i}$, and the contribution column summarizes train-only energetic contribution used for the final 98% gate. Selected residues are displayed first, followed by unselected residues.

## Data Availability

The datasets and source code are publicly available at https://github.com/chemagents/cannabis-biopesticide-mcp-server (accessed on 28 August 2026).

## Supplementary Materials

The following supporting information can be downloaded at https://www.mdpi.com/article/10.3390/ijms27188000/s1.

## Abbreviations

The following abbreviations are used in this manuscript:

AChE	Acetylcholinesterase
CCD	Cannabis Compound Database
GluCl	Glutamate-gated chloride channel
DMPNN	Directed Message Passing Neural Network
RTE	Residue-Term Energy
RMT	Random Matrix Theory

## Appendix A. Random Matrix Theory Filtering of Residue-Term Energy Descriptors

This appendix describes the residue-selection procedure used to construct the mechanistic RTE feature space. The purpose of the procedure was not to use docking scores as direct predictors, but to identify a compact set of amino-acid residues whose residue-level interaction energies carried reproducible signal across training compounds. The approach follows the general use of RMT for cleaning high-dimensional correlation matrices [32,33] and the protein–ligand application proposed by Lee, Brenner, and Colwell [34].

For a given protein, let

$$X_{g}\in R^{n\times p}$$

denote the residue-energy matrix for protein *g*, where rows correspond to ligands and columns correspond to residue-level docking energies. Here and throughout Appendix A, *n* denotes the number of ligands (matrix rows), and *p* denotes the number of residue-level energy features (matrix columns).

$$X_{g}\in R^{n\times p}$$

denote the residue-energy matrix for protein *g*, where rows correspond to ligands and columns correspond to residue-level docking energies. All transformations were fitted on the training rows only. Missing values were replaced with zero after matrix alignment, and each column was centered and scaled using training statistics to obtain $Z_{g}$. The residue correlation matrix was then computed as

$$C_{g}=\frac{Z_{g}^{T}Z_{g}}{n-1}.$$

Eigenvalues and eigenvectors of $C_{g}$ were obtained from

$$C_{g}v_{j}=\lambda_{j}v_{j},$$

with eigenvalues sorted in descending order. Under the null model of a random correlation matrix, the upper edge of the Marchenko–Pastur distribution is [32,33]

$$\lambda_{+}={\left(1+\sqrt{q}\right)}^{2},\;q=\frac{p}{n}.$$

Eigencomponents with $\lambda_{j}>\lambda_{+}$ were treated as signal components, while components within the Marchenko–Pastur bulk were treated as noise-dominated. If no eigenvalue exceeded $\lambda_{+}$, the leading component was retained as a conservative fallback to avoid an empty feature set.

Each residue feature *i* was assigned an RMT score

$$s_{i}=\sum_{j:\lambda_{j}>\lambda_{+}}\lambda_{j}v_{ij}^{2},$$

where $v_{ij}$ is the loading of residue *i* on signal eigenvector *j*. This score is high when a residue contributes strongly to eigencomponents that lie outside the random-noise spectrum. Residues were ranked by decreasing $s_{i}$, producing an ordered list $\pi =(\pi_{1},\pi_{2},\ldots ,\pi_{p})$.

The number of RMT-ranked residues was selected by inner cross-validation on the training set. For each candidate value *m*, a one-dimensional cumulative binding-support descriptor was computed as

$$b_{m}\left(x\right)=\sum_{k=1}^{m}x_{\pi_{k}},$$

where $x_{\pi_{k}}$ is the residue-energy value for residue $\pi_{k}$. Logistic regression was fitted to $b_{m}$ inside StratifiedShuffleSplit folds, and *m* was chosen by the highest mean validation area under the receiver operating characteristic (ROC) area under curve, here denoted as ${\mathrm{AUC}}_{\mathrm{ROC}}$:

$$m^{*}=\underset{m}{arg max}\;{\mathrm{AUC}}_{\mathrm{ROC}}\left(b_{m}\right)$$

The RMT top-$m^{*}$ set was then filtered by train-only energetic contribution. For each residue *i*, the contribution score was defined as

$$c_{i}=\max\left(|{\bar{x}}_{i,\mathrm{active}}\left|,\right|{\bar{x}}_{i,\mathrm{inactive}}|\right),$$

where ${\bar{x}}_{i,\mathrm{active}}$ and ${\bar{x}}_{i,\mathrm{inactive}}$ are the mean residue energies among active and inactive training ligands. Within the RMT top-$m^{*}$ set, residues were sorted by decreasing $c_{i}$. The final selected set was the smallest prefix whose cumulative contribution reached 98%:

$$K^{*}=\min K:\frac{\sum_{k=1}^{K}c_{\eta_{k}}}{\sum_{r\in R_{m^{*}}}c_{r}}\geq 0.98,$$

where $R_{m^{*}}$ is the RMT top-$m^{*}$ set and $\eta_{k}$ is the ordering by decreasing energetic contribution within that set. The selected original residue-energy columns were then used as inputs for the CatBoost docking-consistency model.

This procedure was repeated independently for each outer train/test split and each target protein. RMT decomposition, $m^{*}$ selection, and energetic-contribution filtering used training rows only; held-out test rows were transformed only by retaining the residue columns selected from the corresponding training split. Thus, $m^{*}$ was not chosen from residues shared across all outer splits; instead, recurrence of the same residues across splits was used only as a post hoc stability diagnostic for the selected amino-acid set. This split-aware implementation prevents information leakage from the test set into residue selection.

A representative GLC1 diagnostic illustrating this procedure is shown in Figure A1; it links the Marchenko–Pastur threshold, signal eigencomponents used to compute $s_{i}$, train-only energetic contribution, and the final selected residue set after the 98% gate.
