# Supplementary material for: Biopesticidal Potential of the Cannabis sativa L. Metabolites: A Denoised, Docking-Informed QSAR Model
Source: Int J Mol Sci. 2026 Sep 8;27(18):8000. doi: 10.3390/ijms27188000 (PMC13606784; doi:10.3390/ijms27188000)
Supplement: Supplementary file 1 [file ijms-27-08000-s001.zip › ijms-4488105-supplementary.pdf]

Supplementary Materials

Biopesticidal Potential of the *Cannabis sativa L.* Metabolites: A Denoised, Docking-Informed QSAR Model

Dinara Karamanova [1,*], Nikita Erin [1], Alexander Bodrov [1], Varvara Tkachenko [1,2], Victor Safronov [1,3] and Maxim Fedorov [1,*]

[1] A.A. Kharkevich Institute for Information Transmission Problems, Russian Academy of Sciences, Bolshoy Karetny per. 19, build. 1, Moscow, 127051, Russian Federation
[2] ITMO University, 49 Kronverksky Pr., St. Petersburg, 197101, Russian Federation
[3] Moscow Institute of Physics and Technology, 9 Institutskiy per., Dolgoprudny, Moscow Region, 141701, Russia
* Correspondence: karamanova.da@iitp.ru (D.K.); fedorov@iitp.ru (M.F.)

1. Supplementary Methods

1.1. DMPNN training configuration

Primary prediction of pesticidal activity was performed with a directed message-passing neural network (DMPNN) as implemented in Chemprop. The learned molecular-graph encoding was concatenated with normalized RDKit 2D descriptors before the feed-forward readout, so that the prediction relied simultaneously on automatically extracted structural features and conventional molecular descriptors. The dataset was split randomly into training, validation, and test subsets. Training was terminated by early stopping on the validation objective, and the checkpoint with the best validation score was retained for inference. For each metabolite the model returned the probability of belonging to the pesticide class; compounds with $P \geq 0.70$ were carried forward as candidate biopesticides. The hyperparameters used for the final model are listed in Table S1.

Table S1: Hyperparameters of the DMPNN (Chemprop) model used for primary structure-based prioritization of *C. sativa* metabolites.

Parameter	Value	Description
Message-passing depth	3	Number of directed message-passing steps over the molecular graph
Hidden size	300	Dimensionality of the bond message vectors
Dropout	0.1	Dropout rate applied in message passing and in the readout network
FFN layers	2	Depth of the feed-forward readout network
FFN hidden size	300	Width of the feed-forward readout network
Aggregation	multi	Multi-aggregation of atom messages (mean, sum, and norm)
Additional features	RDKit 2D	Normalized RDKit descriptors concatenated before the readout
LR scheduler	Noam	Linear warm-up followed by exponential decay
Stopping criterion	early stopping	Best-validation checkpoint retained
Output	sigmoid	Probability of belonging to the pesticide class
Decision threshold	$P \geq 0.70$	Cut-off for inclusion in the candidate set

1.2. Performance of the toxicity and ecotoxicity models

The predictive performance of the models used for the toxicological and ecotoxicological characterization of DS1 and DS2 was assessed by random 5-fold cross-validation. Table S2 reports the cross-validated performance for those endpoints that are used in the present study; further endpoints available in the underlying platform but not employed here are not listed. Regression endpoints are summarized by the root-mean-square error (RMSE) in decimal logarithmic units of the corresponding quantity, and classification endpoints by ROC–AUC (one-vs-rest for the multiclass GHS-based aquatic hazard endpoint).

Table S2: Cross-validated performance of the toxicity and ecotoxicity models used in this study. Values are means over five folds of random 5-fold cross-validation.

Endpoint	Metric	Value	Unit
Mouse oral LD_{50}	RMSE	0.45	$\log_{10}$(mg/kg)
Rat oral LD_{50}	RMSE	0.47	$\log_{10}$(mg/kg)
Ames test	ROC–AUC	0.89	—
Carcinogenicity	ROC–AUC	0.79	—
Hepatotoxicity	ROC–AUC	0.81	—
DILI	ROC–AUC	0.90	—
Cardiotoxicity	ROC–AUC	0.93	—
Reproductive toxicity	ROC–AUC	0.74	—
Daphnia magna LC_{50} (48 h)	RMSE	0.82	$\log_{10}$(mmol/L)
96-h Fathead minnow LC_{50}	RMSE	0.72	$\log_{10}$(mmol/L)
Acute aquatic toxicity (57455-2017)	ROC–AUC (OvR)	0.82	—

An RMSE of 0.45–0.47 $\log_{10}$ units for the acute oral LD_{50} models corresponds to a typical prediction error of approximately threefold in mg/kg; for the aquatic endpoints (RMSE 0.72–0.82 $\log_{10}$) the corresponding factor is five- to sevenfold. Predicted LD_{50} and LC_{50} values should therefore be interpreted as order-of-magnitude estimates suitable for relative ranking between compounds rather than as quantitative toxicity values. Among the classification endpoints, reproductive toxicity showed the lowest cross-validated performance (ROC–AUC = 0.74), and its predictions should be treated with corresponding caution.

1.3. Independent open reproduction of the toxicity models

The predictions reported in the main text were retrieved from the Syntelly platform. To make the toxicological part of the study reproducible without access to that platform, the published modelling recipe (Shkil et al., *Molecules* **2024**, *29*, 1826) was independently re-implemented on open data. The reproduction uses a gradient-boosting ensemble (CatBoost, XGBoost, LightGBM and ExtraTrees over ECFP fingerprints, an RDKit fragment panel and RDKit 2D descriptors), trained on the corresponding TOXRIC datasets. The code and the full evaluation output are available at `https://github.com/chemagents/cannabis-biopesticide-mcp-server`.

Table S3 reports the performance of this open reproduction under two splitting schemes. Random 5-fold cross-validation matches the protocol of the original study and allows comparison with the published benchmarks. A Bemis–Murcko scaffold-disjoint cross-validation was run in addition, keeping compounds that share a scaffold within the same fold. The latter is the more demanding setting and is the relevant one when the models are applied to natural products that differ structurally from the training data. The reproduction covers four endpoints. These are the ones for which open training data were available. For the other endpoints we used the platform predictions as retrieved.

Table S3: Performance of the independent open reproduction of the toxicity models under random and Bemis–Murcko scaffold-disjoint 5-fold cross-validation, with the published benchmarks for the same endpoints (Shkil et al., *Molecules* **2024**, *29*, 1826). These models are a reproducibility check and are not the source of the predictions reported in the main text.

Endpoint	Metric	Open (random)	Open (scaffold)	TOXRIC/Syntelly	Shkil et al.
Ames test	ROC–AUC ↑	0.9225	0.877	0.88	0.894
Daphnia magna LC_{50} (48 h)	RMSE ↓	1.026	1.183	1.109	0.817
96-h Fathead minnow LC_{50}	RMSE ↓	0.788	0.947	0.864	0.720
Reproductive toxicity	ROC–AUC ↑	0.586	—	0.927	0.739

Under random splitting the open reproduction matches or exceeds the TOXRIC and Syntelly benchmarks for the Ames, *Daphnia* and fathead minnow endpoints on the same data. Where the original study reports better aquatic metrics, it used roughly twice as many compounds through multi-source aggregation. Reproductive toxicity is the exception: the available set is small ($n = 156$, 88% positives), fold-level scores span a wide range, and the endpoint is reported here as low-confidence.

Scaffold-disjoint validation lowers performance for all three endpoints for which it was run, which is the

expected penalty when structurally related compounds are separated between folds. This gives a more realistic estimate of how such models behave on chemically novel compounds, and it is a further reason to read the metabolome-level toxicological comparison in the main text as indicative rather than quantitative.

1.4. Scaffold-based validation of the DMPNN model

To evaluate the ability of the DMPNN model to generalize to structurally different compounds, an additional paired validation was performed using random and Bemis–Murcko scaffold-based data splitting. In the scaffold-based evaluation, compounds sharing the same Bemis–Murcko scaffold were kept within the same data partition, thereby reducing structural similarity between the training and test compounds.

For this paired comparison, the same DMPNN configuration was evaluated under random and scaffold-based splitting. The random split yielded ROC–AUC = 0.9260 and PR–AUC = 0.9393, whereas the Bemis–Murcko scaffold split yielded ROC–AUC = 0.9084 and PR–AUC = 0.9297. The random-split values differ slightly from those reported in Section 2.3 of the main text, which come from a separate cross-validation run. The results are summarized in Table S4. The moderate decrease under scaffold-based splitting is consistent with the more demanding evaluation on structurally distinct compounds, while the model retained high discriminative performance.

The complete machine-readable results of this paired evaluation are publicly available in the project repository in the file `server/data/reference/dmpnn_stack_rmt_cv.json`. This analysis is reported as an additional generalization test and is separate from the primary random-split performance estimate presented in the main text.

Table S4: Performance of the DMPNN model under random and Bemis–Murcko scaffold-based validation.

Data split	ROC–AUC	PR–AUC
Random split	0.9260	0.9393
Bemis–Murcko scaffold split	0.9084	0.9297

1.5. Model applicability coverage

Table S5 reports the share of compounds falling outside the applicability domain of each endpoint model. Coverage is uneven across endpoints. For most endpoints fewer than 15% of DS1 compounds are outside the domain. Cardiotoxicity is the exception, with about two thirds outside, and its predictions were therefore not used for comparative interpretation.

Table S5: Compounds outside the applicability domain of each toxicity and ecotoxicity model.

Endpoint	DS1, *n*	DS1, %
Mouse oral LD_{50}	235	4.7
Rat oral LD_{50}	203	4.0
Ames test	198	3.9
Carcinogenicity	180	3.6
Hepatotoxicity	261	5.2
DILI	165	3.3
Cardiotoxicity	3290	65.5
Reproductive toxicity	693	13.8
Daphnia magna LC_{50} (48 h)	684	13.6
96-h Fathead minnow LC_{50}	724	14.4
Acute aquatic toxicity	725	14.4

2. Supplementary Figures

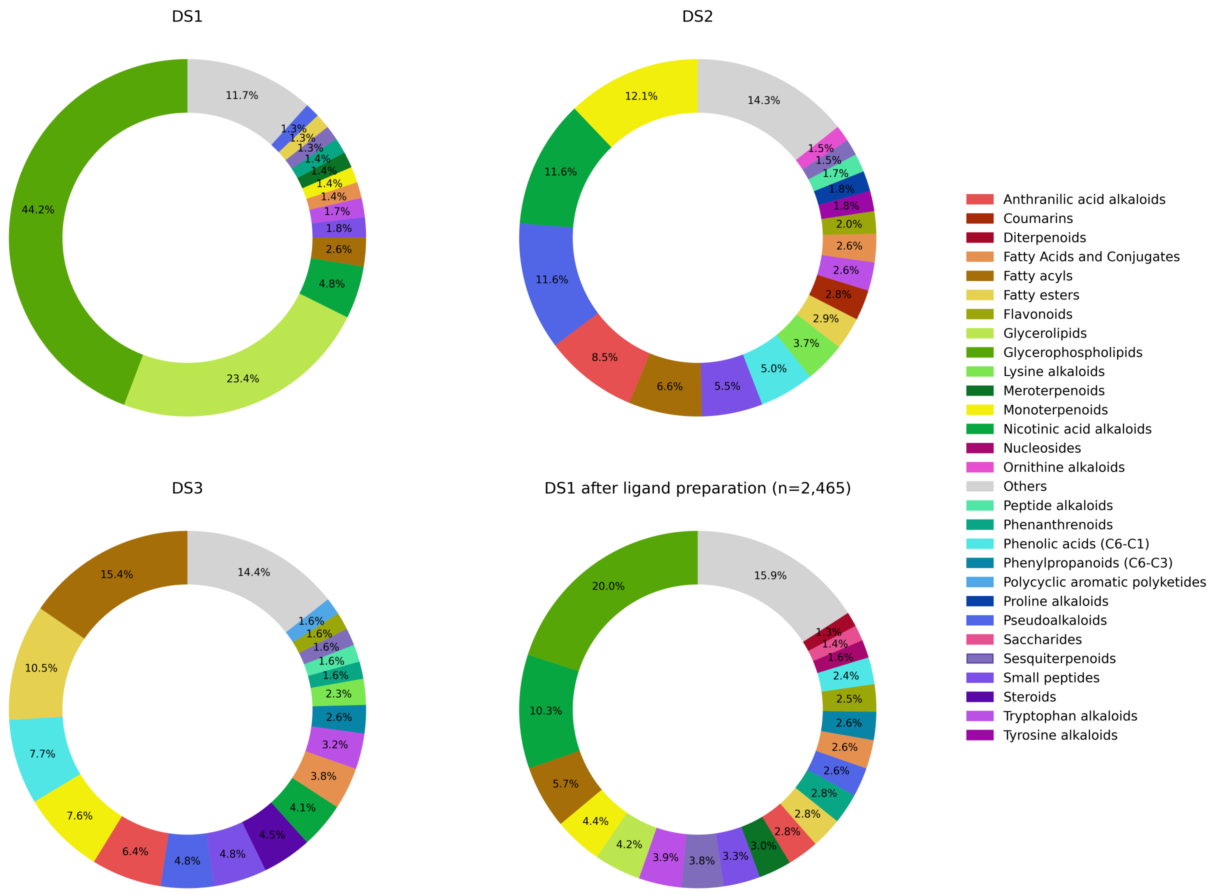

Figure S1: Distribution of chemical superclasses across the compound datasets and the docking-prepared subset: standardized *C. sativa* metabolites (DS1); registered pesticides (DS2); inactive reference compounds (DS3); and DS1 compounds successfully retained after ligand preparation for molecular docking (n = 2,465). Superclasses accounting for less than 1.3% of a set were merged into "Others". Structural classification was performed with NPClassifier.

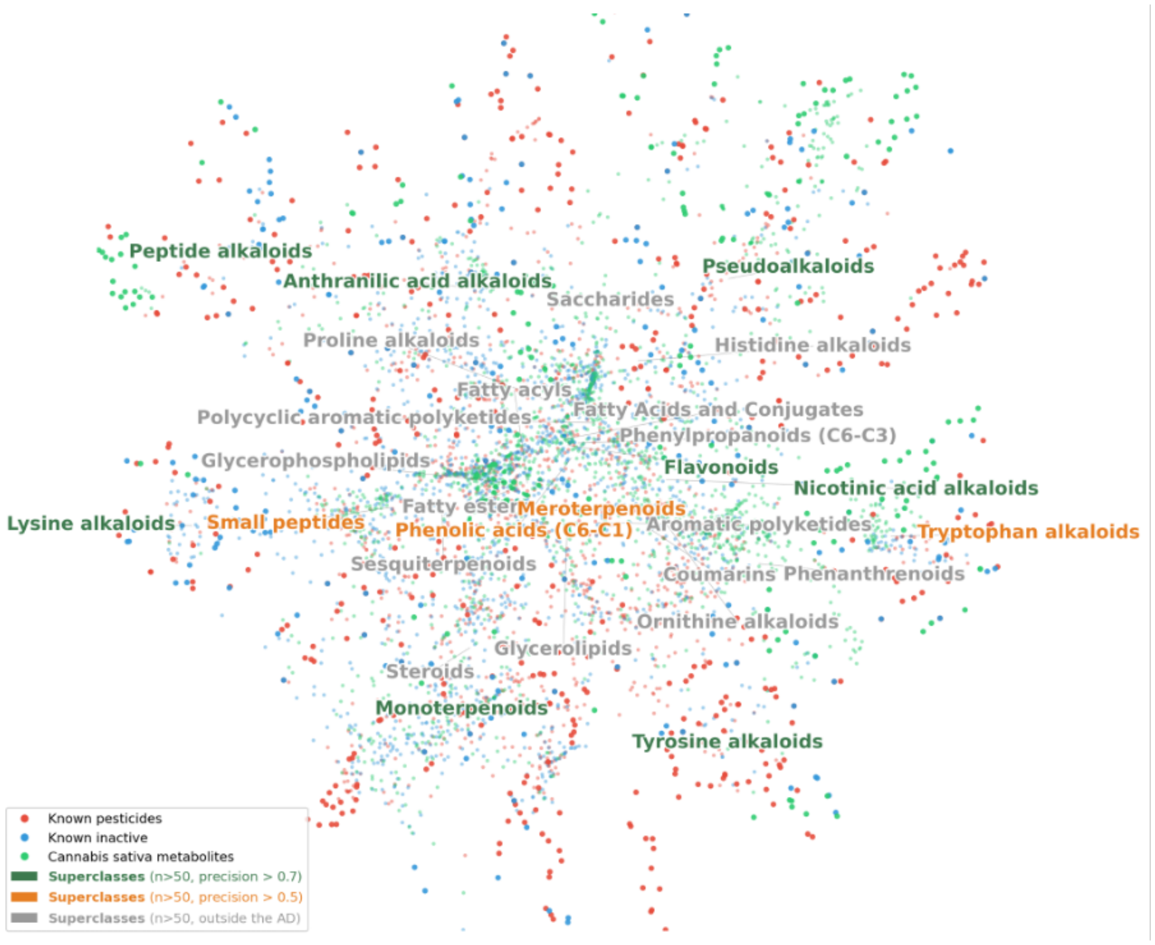

Figure S2: t-SNE visualization of the chemical space spanned by DS1, DS2 and DS3. Each point corresponds to an individual compound, coloured by its NPClassifier superclass. Labels mark the most populated superclasses; label colour encodes the mean model confidence within the corresponding superclass.
